# Monocyte-to-Neutrophil Ratio as an Immunological Marker of Left Ventricular Hypertrophy in Children with Primary Hypertension

**DOI:** 10.3390/jcm14113896

**Published:** 2025-06-01

**Authors:** Katarzyna Dziedzic-Jankowska, Radosław Pietrzak, Michał Szyszka, Adam Bujanowicz, Anna Stelmaszczyk-Emmel, Bożena Werner, Piotr Skrzypczyk

**Affiliations:** 1 Department of Pediatrics and Nephrology, Medical University of Warsaw, 02-091 Warsaw, Poland; katarzyna_dziedzic11@wp.pl; 2 Department of Pediatric Cardiology and General Pediatrics, Medical University of Warsaw, 02-091 Warsaw, Poland; rpietrzak@wum.edu.pl (R.P.); bozena.werner@wum.edu.pl (B.W.); 3 Department of Pediatrics and Nephrology, Doctoral School, Medical University of Warsaw, 02-091 Warsaw, Poland; mszyszka@wum.edu.pl (M.S.); abujanowicz@wum.edu.pl (A.B.); 4 Department of Laboratory Diagnostics and Clinical Immunology of Developmental Age, Medical University of Warsaw, 02-091 Warsaw, Poland; anna.stelmaszczyk-emmel@wum.edu.pl

**Keywords:** primary hypertension, children, subclinical inflammation, monocyte-to-neutrophil ratio, left ventricular mass index, left ventricular hypertrophy

## Abstract

**Background/Objectives**: Activation of the immune system and subclinical inflammation participate in the pathogenesis of primary hypertension (PH) and the formation of hypertension-mediated organ damage. Our study aimed to investigate the relationship between subclinical inflammation and left ventricular hypertrophy (LVH) in pediatric patients with PH. **Methods**: In 34 untreated children with PH (15.1 ± 2.1 years, 28 boys, 6 girls), we investigated markers of subclinical inflammation (high-sensitivity CRP, interleukin 18, and complete blood count-derived indices), parameters of the left ventricle from 2D-echocardiography, office and ambulatory blood pressure, and selected clinical and biochemical parameters. **Results**: LVH was revealed in 12 (35.3%) patients, and abnormal relative wall thickness (RWT) was found in 6 (17.6%) children. Left ventricular inner dimension at end diastole (LVEDd) Z-score correlated negatively with neutrophils (r = −0.583, *p* = 0.001), neutrophil-to-lymphocyte ratio (NLR) (r = −0.562, *p* = 0.002), and positively with monocyte-to-neutrophil ratio (MNR) (r = 0.605, *p* = 0.001) and left ventricular mass (LVM) for lean body mass Z-score, while LVMI [g/m^2^] correlated positively with MNR (r = 0.495, *p* = 0.005 and r = 0.433, *p* = 0.011). RWT correlated positively with neutrophil count (r = 0.356, *p* = 0.039 and r = 0.347 *p* = 0.044) and with monocyte count (r = 0.378, *p* = 0.027 and r = 0.365, *p* = 0.034). Patients with LVH had significantly lower NLR (1.430 ± 0.409 vs. 1.797 ± 0.521, *p* = 0.043) and higher MNR ratios (0.171 ± 0.031 vs. 0.144 ± 0.037, *p* = 0.042). The receiver operating characteristic analysis demonstrated good diagnostic profiles for mean platelet volume (MPV), NLR, and MNR as predictors of LVH. In multivariate analysis, MNR was the only significant predictor of LVH (OR: 1.329, 95CI: 1.007–1.756). **Conclusions**: Monocyte-to-neutrophil ratio may be an easily accessible marker of left ventricular hypertrophy in children with primary hypertension.

## 1. Introduction

Arterial hypertension (AH) is found in approximately 4.0% of pediatric patients [1]. Though AH is extremely rare in infants, its prevalence rises alongside children’s age, reaching even 8–10% at the age of 18 [2]. The problem of arterial hypertension in the population appears to be even greater in the post-pandemic COVID-19 era [3]. Traditionally, secondary forms of hypertension (e.g., renal, renovascular, or hormonal) were considered as leading causes of elevated blood pressure in the developmental period. Still, nowadays it is known that primary (i.e., essential) hypertension might cover as many as half of these cases [4]. Primary hypertension (PH) is a complex disease with still unclear pathogenesis [5]. It is hypothesized that subclinical, low-grade inflammation might play a vital role in the pathogenesis of PH in all age groups [6,7]. According to experimental data, an increase in blood pressure damages the endothelium and releases different neo-antigens, which, in turn, trigger the activation of both the innate and acquired immune systems [8].

There are numerous ways to measure subclinical (low-grade) inflammation. The serum concentration of interleukins (e.g., interleukin 6 or 18), high-sensitivity C-reactive protein (hs-CRP), or even simple, complete blood count-derived markers like neutrophil count, neutrophil-to-lymphocyte ratio (NLR), platelet count, platelet-to-lymphocyte ratio (PLR), and platelet volume (MPV) have been used in numerous studies as robust markers of subclinical inflammation in children and adults with primary hypertension [9,10,11,12].

Hepatocytes produce CRP in response to different proinflammatory stimuli. CRP binds lysophosphatidylcholine on the bacterial membrane and activates the complement system [13]. In numerous epidemiological adult studies, CRP was an independent predictor correlating with the risk of hypertension, atherosclerosis, and cardiovascular events [14]. Notably, pediatric studies, e.g., the manuscripts of Trojanek [15], Wasilewska [16], Hou [17], and our group [18], revealed that serum hs-CRP concentrations were significantly higher in hypertensive patients than in their healthy peers.

Interleukin 18 belongs to the interleukin 1 superfamily and is produced mainly by macrophages. IL-18 is a proinflammatory cytokine that acts on T and NK cells to stimulate interferon γ production. Of note is that IL-18 and its receptors are expressed on endothelial cells and vascular smooth muscle cells, thus suggesting its role in vascular diseases [19]. Yamagami and Rabkin found that serum IL-18 concentrations were higher in hypertensive adults than in those with normal blood pressure [11,12].

The white blood cells (WBCs), their subtypes, and platelets are the essential players in the inflammatory cascade. Therefore, direct counts of white blood cell subtypes and their ratios, like NLR, MLR, MNR, and PLR, have been intensively studied in various inflammatory and cardiovascular diseases. In adults, they correlate with numerous cardiovascular diseases like atherosclerosis, heart failure, acute coronary syndromes, and arterial hypertension [10,20]. Interestingly, pediatric patients with PH were found to have higher counts of white blood cells [17], neutrophils [17,18], monocytes [18], and platelets [16,21]; larger platelets [16]; and higher NLR [22], PLR [22], and MLR [22] than their healthy peers.

Hard endpoints like myocardial infarction, stroke, and death from cardiovascular causes are virtually nonexistent in adolescent patients with primary hypertension. Nevertheless, subclinical hypertension-mediated organ damage (HMOD) is found in up to half of pediatric patients with primary hypertension [23]. Two large-scale central European studies published over 15 years ago unmasked a high prevalence of cardiac damage in children with arterial hypertension [24,25]. A meta-analysis of 38 pediatric studies showed that arterial hypertension significantly increases the risk for left ventricular hypertrophy (LVH) and elevated markers of arterial damage (aortic pulse wave velocity and common carotid artery intima-media thickness) [26]. A recently published meta-analysis revealed that LVH is found in 30.5% of children and young adults with PH, with a predominantly eccentric LVH pattern. Increased BMI was the most significant risk association for LVH in hypertensive youth [27]. Another meta-analysis by the same group showed that hypertensive children and adolescents presented with signs of hyperkinetic function of the left ventricle and demonstrated evidence of increased left ventricular strain and impaired diastolic function compared to normotensive controls [28].

Single data in pediatric patients suggest that inflammatory markers may be associated with diastolic dysfunction [17] and arterial stiffness in children with PH [21].

Our study aimed to evaluate the relationship between left ventricular parameters and selected inflammatory markers in untreated adolescents with primary hypertension.

## 2. Materials and Methods

This was a cross-sectional, single-center, retrospective study. Initially, we included all the patients hospitalized in our pediatric nephrology ward due to suspicion of arterial hypertension from 2017 to 2021. The inclusion criterion was arterial hypertension diagnosed according to the European Society of Hypertension guidelines [29] and confirmed by ambulatory blood pressure monitoring (ABPM). The exclusion criteria were lack of consent to participate in the study, acute (e.g., common infections) and chronic inflammatory conditions (e.g., autoimmune diseases), known allergic diseases, chronic kidney disease, congenital or acquired heart defects or heart failure, secondary hypertension, and pharmacological antihypertensive treatment or treatment influencing immunological system (e.g., corticosteroids). The baseline data of 56 children with PH were presented in the recently published article [18]. Among these patients, we included 34 patients with echocardiography performed within 1 month of the analysis of inflammatory markers.

The researchers obtained approval from the local bioethics committee to conduct the study (approval No. KB/58/2016, 15 March 2016, amendment No. KB/53/A2023, 12 June 2023). All procedures involving human participants followed the highest ethical standards of the institutional research committee and were performed in accordance with the Declaration of Helsinki on the treatment of human subjects and its later amendments. All participants and their legal representatives signed informed consent forms before entering into the study.

In the study participants, we measured the following clinical parameters: age, sex (male/female), AH vintage, duration of pregnancy, and birth weight, height, weight, and body mass index (BMI) [30]. Overweight and obesity were defined according to World Health Organization definitions: BMI ≥ 85th and <95th percentile, and ≥95th percentile, respectively [31].

Office systolic and diastolic blood pressure (SBP, DBP) were measured with a Welch Allyn VSM Patient Monitor 300 (Welch Allyn Inc., Skaneateles Falls, NY, USA) [mmHg] according to the ESH 2016 guidelines [29] and were expressed as [mm Hg] and as Z-scores [32]. Office pulse pressure (PP) was defined as the difference between systolic and diastolic blood pressure. A Suntech Oscar 2 oscillometric device (SunTech Medical, Inc., Morrisville, NC, USA) was used to evaluate 24 h blood pressure parameters with assessment of the following parameters [33]: systolic, diastolic, and mean blood pressure over the course of 24 h (SBP, DBP, MAP, 24 h) expressed as [mm Hg] and Z-scores [33], systolic and diastolic blood pressure loads (SBPL, DBPL) over the course of 24 h expressed as [%], and systolic and diastolic blood pressure dipping (SBP DIP, DBP DIP) defined as a difference between mean daytime blood pressure and mean nighttime blood pressure expressed as a percentage of the daytime value.

Echocardiography was performed using a Philips iE33 device and an S5-1 transducer (Philips, Amsterdam, The Netherlands). Left ventricle dimensions were calculated using M-mode assessment of the left ventricle with simultaneous recording of ECG in the second limb lead. The detailed methodology was described in [34,35]. The following parameters were collected in the end-diastolic phase: the interventricular septum thickness at end diastole (IVSd) (mm), left ventricular inner dimension at end diastole (LVEDd) (mm), and left ventricular posterior wall at end diastole (LVPWd) (mm). Z-scores for IVSd, LVEDd, and LVPWd were calculated from normative data by Overbeek et al. [36]. Left ventricular mass (LVM) (g) was calculated from the Devereux equation [37]. LVM was indexed by body surface area [g/m^2^] [37], by the DeSimone formula (g/m^2.7^) [38], and by the formula proposed by Chinali et al. (g/m^2.16^) [39]. Also, the Z-scores of left ventricular mass for lean body mass [40] and height [31] were calculated. Left ventricular hypertrophy (LVH) was defined as LVMI (g/m^2^) ≥ 115 g/m^2^ in boys or LVMI (g/m^2^) ≥ 95 g/m^2^ in girls [37] or LVMI (g/m^2.7^) ≥ 95c. for sex and age [41]. Relative wall thickness (RWT) was defined in three ways: 2 × LVPWd/LVEDd, 2 × IVSd/LVEDd, and (IVSd + LVPWd)/LVEDd. Abnormal RWT was defined as RWT ≥ 0.42 [37].

The level of subclinical inflammation was evaluated using serum inflammatory indices and complete blood count-derived mediators. The methodology was described in our previous study [18]. We performed complete blood count (CBC) using a Sysmex XN1000 hematologic analyzer (Sysmex Corporation, Kobe, Japan) and assessed the following inflammatory markers: neutrophils (NEU; 1000/μL), lymphocytes (LYM; 1000/μL), platelets (PLT; 1000/μL), mean platelet volume (MPV; fL), and neutrophil-to-lymphocyte, platelet-to-lymphocyte, monocyte-to-lymphocyte, monocyte-to-neutrophil, and platelet-to-mean platelet volume ratios (NLR, PLR, MLR, MNR, PMPVR). Blood for hs-CRP and IL18 measurement was allowed to clot for 30 min, was then centrifuged, and the obtained serum was frozen at −80 °C. The concentrations of hs-CRP (mg/L) and IL18 (pg/mL) were determined by the enzyme-linked immunosorbent assay method (DRG^®^ CRP, HS C-Reactive Protein Catalog Number EIA-3954, DRG International Inc., Springfield, NJ, USA, and Human IL-18 ELISA Kit, Catalog Number BMS267-2, ThermoFisher Scientific, Austria, Vienna) using a Biochrom Asys UVM 340 Scanning Microplate Reader (Biochrom Ltd., Cambridge, UK).

The remaining biochemical parameters were evaluated by standard local laboratory methods (dry chemistry (VITROS 5600, Ortho Clinical Diagnostics, New Jersey, USA)): serum creatinine (mg/dL), urea (mg/dL), uric acid (mg/dL), and lipidogram: total, high-density lipoprotein (HDL), and low-density lipoprotein (LDL) cholesterol (mg/dL), triglycerides (mg/dL), and morning urinary albumin–creatinine ratio (mg/g). Vitamin D (25OHD) (ng/mL) was assessed by chemiluminescence (Alinity ci, Abbott Laboratories, Lake Bluff, IL, USA), and normal values of vitamin D concentrations were taken from local recommendations [42]. In all participants, we calculated the estimated glomerular filtration rate (GFR_S_) according to the Schwartz formula [43]. An albumin–creatinine ratio ≥ 30 mg/g was considered abnormal [29], and uric acid > 5.5 mg/dL was considered elevated, according to Feig [44]. Normative values for pediatric lipid parameters were taken from Stewart [45].

Statistical data were analyzed using Dell Statistica 13.0 PL software (TIBCO Software Inc., Palo Alto, CA, USA). Heatmaps was generated in Python (version 3.12). The sample size estimated based on the available literature, with a statistical power of 0.8, *p* = 0.05, and an effect size of 0.50, should be at least 30. The normality of data distribution was analyzed using the Shapiro–Wilk test. Data were reported as absolute numbers, the mean ± standard deviation (SD), and the interquartile range (IQR). The following tests were used (depending upon variables’ distribution): Student’s *t*-test, Mann–Whitney U test, Kruskal–Wallis test, Fisher’s exact test, Pearson’s linear correlation, Spearman’s rank correlation, chi-square test, Fisher’s exact test, and the receiver operating characteristic (ROC) analysis. Multivariate analysis of factors related to left ventricular hypertrophy was performed using logistic regression. Parameters that differentiated groups with and without LVH, with *p* below 0.10, were included in the model. A *p*-value below 0.05 was considered statistically significant.

## 3. Results

The clinical and biochemical results in the study group are depicted in Table 1. In the study group, three-quarters of the patients were boys, and the average age in the study group was about 15 years. The average duration of hypertension was about 13 months. Also, three-quarters of the patients were overweight or obese. All patients had normal renal function. Fifteen patients had borderline or elevated total cholesterol, eight had borderline or elevated LDL cholesterol, and fourteen had borderline or reduced HDL cholesterol. Elevated or borderline triglyceride concentrations were found in 19 children. Twenty-one children had elevated uric acid concentration. As for 25OHD supply, 4 children had severe deficiency, 17 had deficiency, 10 had insufficient supply, and only 3 patients had adequate vitamin D concentration. Abnormal urinary albumin excretion was found in only one adolescent (34.5 mg/g). The inflammatory markers are shown in Table 2.

Table 3 presents office and ambulatory blood pressure in the studied children. Based on ABPM results at the time of the study, isolated systolic hypertension was found in 28 (82.4%) patients and systolic–diastolic hypertension in the remaining 6 (17.6%) subjects; 9 (26.5%) patients were extreme dippers, 12 (35.3%) were dippers, and 13 (38.2%) were non-dippers.

Table 4 shows echocardiographic parameters in the studied children. Left ventricular hypertrophy was revealed in 12 (35.3%) patients. Abnormal relative wall thickness was found in 6 (17.6%) children. Two out of these six patients also had left ventricular hypertrophy.

**Figure 1:**
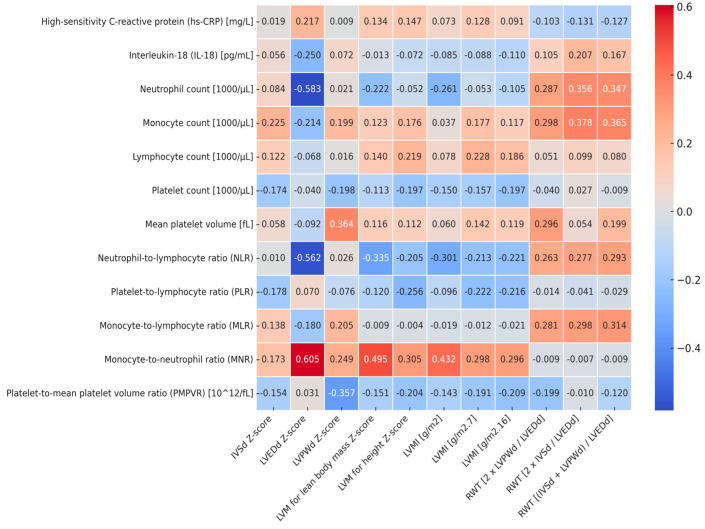
Heatmap of correlations between markers of left ventricular hypertrophy, relative wall thickness, and markers of subclinical inflammation. IVSd—the interventricular septum thickness at end diastole, LVEDd—left ventricular inner dimension at end diastole, LVPWd—left ventricular posterior wall at end diastole, LVM—left ventricular mass, LVMI—left ventricular mass index, RWT—relative wall thickness.

**Figure 2:**
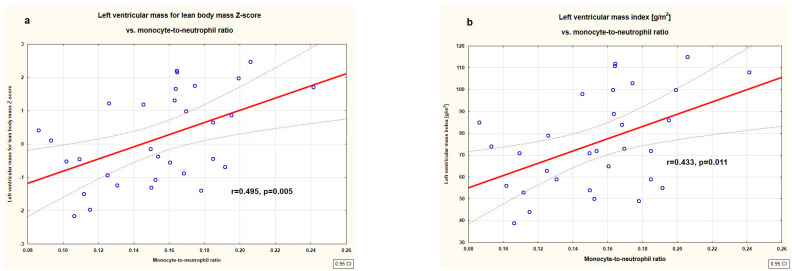
Correlations between left ventricular mass for lean body mass Z-score (**a**) and left ventricular mass index [g/m^2^] (**b**) with monocyte-to-neutrophil ratio in the studied untreated children with primary hypertension.

Figure 1 (heatmap) shows correlations between markers of left ventricular hypertrophy, relative wall thickness, and markers of subclinical inflammation. LVEDd Z-score correlated negatively with neutrophil count (r = −0.583, *p* = 0.001) and NLR (r = −0.562, p=0.002) and positively with MNR (r = 0.605, *p* = 0.001). LVM correlated positively with lean body mass Z-score, and LVMI [g/m^2^] correlated positively with MNR (r = 0.495, *p* = 0.005 and r = 0.433, *p* = 0.011, respectively). In addition, relative wall thickness expressed as 2 × IVSd/LVEDd and as (IVSd + LVPWd)/LVEDd correlated positively with both neutrophil count (r = 0.356, *p* = 0.039 and r = 0.347 *p* = 0.044, respectively) and with monocyte count (r = 0.378, *p* = 0.027 and r = 0.365, *p* = 0.034, respectively). No other significant correlations were found between markers of left ventricular hypertrophy and inflammatory indices. The linear correlations between LVM for body mass Z-score, LVMI [g/m^2^], and MNR were presented in Figure 2.

In the studied children, we found no significant correlations between BMI, BMI Z-score, and markers of left ventricular hypertrophy (Supplementary Table S1). We found only negative correlations between BMI, BMI Z-score, and LVEDd Z-score (r = −0.463, *p* = 0.015 and r = −0.540, *p* = 0.004, respectively), and a positive correlation between BMI and LVPWd (r = 0.362, *p* = 0.035). BMI Z-score correlated positively with neutrophil count (r = 0.427, *p* = 0.012) and negatively with PLR (r = −0.375, *p* = 0.029) and MNR (r = −0.388, *p* = 0.023). We analyzed the impact of obesity on our results. We compared obese (*n* = 17) and non-obese (normal and overweight) patients (*n* = 17). First of all, the obese patients had higher neutrophil count (4.7 ± 1.3 vs. 3.2 ± 0.8 [1000/µL], *p* = 0.0004), higher NLR (1.887 ± 0.529 vs. 1.449 ± 0.395, *p* = 0.010), lower MNR (0.134 ± 0.033 vs. 0.173 ± 0.031), and lower HDL-cholesterol (45.4 ± 10.1 vs. 55.7 ± 16.7 [mg/dL], *p* = 0.038) compared to overweight/normal-weight patients. There was no difference between the groups regarding blood pressure and LV parameters except for a lower LVEDd Z-score in the obese patients (−1.671 ± 0.387 vs. −0.490 ± 1.070, *p* < 0.001). In the non-obese group, we found the following correlations: LVEDd vs. neutrophil count (r = −0.557, *p* = 0.020), NLR (r = −0.483, *p* = 0.050), and MNR (r = 0.485, *p* = 0.049); LVEDd Z-score vs. hcCRP (r = 0.530, *p* = 0.029), neutrophil count (r = −0.652, *p* = 0.005), NLR (r = −0.531, *p* = 0.028), and MNR (r = 0.592, *p* = 0.012); LVMI [g/m^2^] vs. MNR (r = 0.488, *p* = 0.047); LVM for lean body mass Z-score vs. MNR (r = 0.558, *p* = 0.020). On the contrary, in the obese patients, we found only correlations between LVMI [g/m^2.7^] and lymphocyte count (r = 0.511, *p* = 0.036) and between RWT (IVSd + LVPWd)/LVEDd) and monocyte count (r = 0.483, *p* = 0.050). No correlations between MNR and LV parameters were revealed in obese patients.

**Figure 3:**
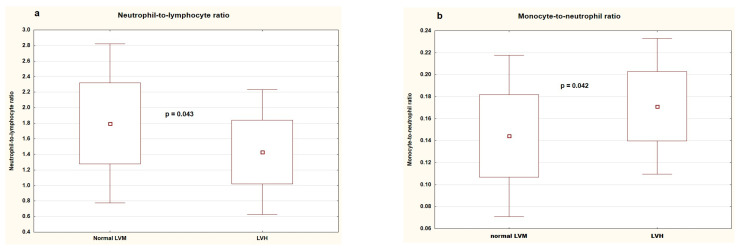
Neutrophil-to-lymphocyte ratio (**a**) and monocyte-to-neutrophil ratio (**b**) in patients without and with left ventricular hypertrophy. LVM—left ventricular mass, LVH—left ventricular hypertrophy.

Table 5 shows clinical and biochemical parameters, blood pressure, and inflammatory markers in patients with and without left ventricular hypertrophy. There was no difference between the groups regarding age, sex, BMI, duration of gestation, birth weight, duration of hypertension, uric acid, lipids, vitamin D, and urinary albumin loss. The groups did not differ significantly in office and ambulatory blood pressure. As for inflammatory markers, patients with LVH had significantly lower neutrophil-to-lymphocyte ratios (1.430 ± 0.409 (IQR: 1.112–1.790) vs. 1.797 ± 0.521 (IQR: 1.455–2.140), *p* = 0.043) and higher monocyte-to-neutrophil ratios (0.171 ± 0.031 (IQR: 0.154–0.187) vs. 0.144 ± 0.037 (IQR: 0.112–0.178), *p* = 0.042) (Figure 3), and there was a trend towards a higher mean platelet volume (11.042 ± 1.324 (IQR: 10.7–11.9) vs. 10.114 ± 1.365 (IQR: 9.2–11.1), *p* = 0.042).

We performed an ROC analysis for MPV, NLR, and MNR. All three markers demonstrated good diagnostic profiles (area under the curve, sensitivity, and specificity) as predictors of left ventricular hypertrophy (Table 6). Also, multivariate analysis using logistic regression revealed that MNR was the only significant predictor of left ventricular hypertrophy (OR: 1.329, 95% CI: 1.007-1.756).

## 4. Discussion

Our single-center, cross-sectional study analyzed the relationship between inflammatory markers and markers of left ventricular hypertrophy in a group of untreated children with primary hypertension. We showed that both left ventricular end-diastolic dimension and differentially expressed left ventricular mass correlated significantly positively with the monocyte-to-neutrophil ratio. In addition, relative wall thickness positively correlated with direct counts of neutrophils and monocytes. A higher MNR ratio characterized patients with left ventricular hypertrophy. ROC analysis and logistic regression analysis confirmed the usefulness of the MNR index as a marker of left ventricular hypertrophy in untreated children with primary hypertension.

Hypertension-mediated organ damage is found in up to 50% of children with primary hypertension at the time of diagnosis [23]. The heart and arteries, as organs directly exposed to high blood pressure, are the first to be affected by lesions. Increased blood pressure leads to changes in the heart, which are initially adaptive–there is hypertrophy of cardiomyocytes and thickening of the heart walls. The systolic function is maintained for a long time, and much earlier, diastolic dysfunction develops [28]. We found the prevalence of LVH in about one-third of untreated children with PH. Our results are close to those of a recently published meta-analysis including 5620 children. The authors showed the presence of LVH in 30.5%. In the meta-regression performed by the authors, the risk factor for LVH was the BMI Z-score, accounting for 41% of the observed heterogeneity [27]. Our single-center study found no correlation between left ventricular hypertrophy or left ventricular mass indexed differently and BMI or BMI Z-score. We cannot exclude that the lack of such a relationship was due to a much smaller number of patients than in the meta-analysis already cited [27]. It is worth noting the relatively long duration of hypertension in our group (about 13 months on average), which may have influenced the presence of HMOD, including LVH. Patients had not previously received pharmacological treatment, nor had they received a full structured non-pharmacological approach.

Many hemodynamic, endocrine, paracrine, and autocrine factors are involved in the development of left ventricular hypertrophy. The immune mechanisms involved in developing PH and HMOD have been intensively studied for over 20 years. It has been hypothesized that left ventricular hypertrophy might also be an inflammatory condition [46]. Immune cells and released cytokines participate in the pathogenesis of cardiovascular remodeling. They eliminate necrotic bodies and cells and promote angiogenesis and scar repair. Proinflammatory cytokines stimulate cardiomyocyte hypertrophy and, above all, myocardial fibrosis. The immune system acts here directly and through interplay with the renin–angiotensin–aldosterone and the sympathetic system. Studies have shown that angiotensin II and excessive catecholamines lead to the formation of effector-like T cells that infiltrate the myocardium. On the other hand, activation of the immune system causes an increase in the production of angiotensin II and aldosterone, creating a vicious circle of immune–endocrine mechanisms leading to LVH [47].

We can assume that primary hypertension was the only factor affecting left ventricular mass and immune status in our group. We excluded children with known heart pathologies or acute or chronic inflammatory conditions. Also, none of the participants received antihypertensive or immunosuppressive medications.

Numerous adult studies have shown correlations between hs-CRP and left ventricular mass or risk for left ventricular hypertrophy [46,48,49]. The same associations were found, among others, in patients with resistant hypertension [50] or those with end-stage kidney disease treated with hemodialysis [51]. Interestingly, two pediatric studies by Assadi confirm this positive association between CRP and LVH [52,53]. A decrease in CRP concentration might also be connected with the regression of LVH. For 12 months, Litwin et al. observed 86 children with primary hypertension and revealed that those with an LVMI decrease also had a higher hs-CRP drop. Nevertheless, in a multivariate analysis, reduction in waist circumference was the main predictor of LVMI decrease, whereas reduction in hs-CRP strongly predicted arterial damage regression [54]. Our cross-sectional study did not show any significant correlations between hs-CRP and markers of left ventricular hypertrophy. Potential reasons for the lack of such correlation in our cohort include a different method of determination (in our case, ELISA; in Assadi’s studies, the nephelometric method [52,53]; in Litwin’s study, immunoturbidimetric [54]) and a small group. Large prospective observational and interventional studies are indeed necessary to determine the role of hs-CRP as a marker of heart damage in pediatric patients with primary hypertension.

Turkish authors found that serum L-18 levels independently predicted LVMI in the general population and newly diagnosed hypertensive patients [55]. Authors from Egypt, studying a group of 50 children with end-stage kidney disease (ESKD) treated with hemodialysis, showed that IL-18 concentration (next to hs-CRP) was an independent determinant of LVH [56]. Again, as in the case of hs-CRP, the lack of correlation in our cohort may be due to a different disease duration, the use of kits from other manufacturers, or, as in the case of the Egyptian study, a more severe inflammation in dialysis children.

Adult studies revealed that complete blood count-derived markers correlated with left ventricular mass and risk of LVH. Associations with target organ damage have been shown for leukocytes, platelets, and even red blood cell parameters [57]. Chinese authors retrospectively analyzed large cohorts of adult patients with PH and found that NLR was an independent predictor for LVH [49,58]. Hou found no correlations between these indices and left ventricular mass in children with PH but revealed that elevated neutrophil count and NLR might be markers of diastolic dysfunction [17]. Our study compared patients without and with LVH; the results showed higher NLR index values in the former group. It is difficult to explain this observation unequivocally. However, it should be emphasized that correlations and multivariate analysis did not confirm it. As for other markers, larger studies, as well as prospective ones, would be advisable to assess the usefulness of the NLR index in predicting changes in the heart in children with PH.

Large platelets exert more proinflammatory and prothrombotic actions than small platelets [59]. Mean platelet volume (MPV) is an easy marker of platelet activation, and elevated MPV is closely associated with cardiovascular diseases [60,61,62]. In some adult studies, MPV was an independent predictor of left ventricular mass index [61,62], while in others, no such relationship was found [63,64]. There are scarce data on the relationship between MPV and HMOD in children. In their study analyzing 42 children with ESKD, authors from Iran found that MPV values were significantly higher in the subjects than in the control group. In addition, in ESKD children, MPV correlated positively with LVMI and inversely with ejection fraction [65]. Conversely, no correlation between LVM, arterial damage, and MPV was found in a Turkish cohort of 60 obese children [66]. Also, our previous study showed no association between MPV and parameters of arterial damage in patients with PH [21]. However, in this study, we showed a trend towards higher MPV values in children with LVH, and the cut-off point for increased risk of LVH in the ROC analysis was 10.6 fl. MPV is a simple, available parameter that could potentially serve as a marker of LVH in children with primary hypertension.

The monocyte-to-neutrophil ratio (MNR) is another relatively new CBC-derived marker of inflammation, and data on its usefulness are only accumulating. It has been shown to have prognostic significance in adults, including diabetic foot ulcers [67], chronic obstructive pulmonary disease [68], COVID-19 infection [69], renal cell carcinoma [70], and acute myeloid leukemia [71]. To our knowledge, only three studies have been published to date analyzing MNR in the context of cardiovascular disease. Our previous study did not demonstrate any differences in MNR between children with PH, white coat hypertension, and healthy children [18]. In turn, Japanese authors, in a study of adult patients with acute aortic dissection, demonstrated that the MNR index was higher in patients with cardiac tamponade [72]. Chinese authors, in a study on patients after acute ischemic stroke treated with intravenous thrombolysis, showed that the MNR value was lower in patients after myocardial infarction compared to the control group and that a low MNR value 3 months after stroke was a risk factor for poor prognosis [73]. A potential mechanism linking the MNR index with left ventricular hypertrophy is the involvement of monocytes and macrophages in the repair processes and remodeling of the left ventricle [74]. It is believed that one of the mechanisms of the frequent occurrence of left ventricular hypertrophy in primary aldosteronism is the stimulation of macrophages by aldosterone to produce pro-inflammatory and pro-fibrotic cytokines such as transforming growth factor β1 [75]. In a recently published study, authors from China revealed, in a mouse model using machine learning, six hub monocyte/macrophage-related genes (*Ankrd1, Birc5, Nuf2, C1qtnf6, Fcgr3,* and *Cdca3*) that accurately predicted hypertensive LVH [76].

In this study, the MNR index was the best immunological marker of left ventricular hypertrophy. The MNR value correlated positively with LVEDd Z-score, LVM for lean body mass Z-score, and LVMI [g/m^2^]. The MNR value was significantly higher in patients with LVH. For MNR, a good diagnostic profile was demonstrated as a predictor of LVH. Finally, multivariate analysis using logistic regression revealed that MNR was the only significant predictor of LVH in this model. Although we did not show an effect of BMI values on left ventricular mass, we showed a negative correlation between BMI and MNR. Moreover, very interestingly, in the group of obese patients, there was no correlation between MNR and left ventricular mass parameters. Obese patients are characterized by a higher severity of inflammation regardless of blood pressure values [77]. It cannot be excluded that in obese patients, other factors shape left ventricular mass, and the relationship between inflammation and HMOD is different. Our cross-sectional study indicates the need for further research on this marker. We plan to extend the study to a larger group of patients and correlate the MNR index with arterial damage. The particular strengths of our study are the analysis of numerous inflammatory markers and a vast number of left ventricular mass indices related to well-established normative values. Before starting the study, we performed a thorough literature review and selected virtually all CBC-derived inflammatory markers, including new and poorly studied ones, such as MNR. All echocardiographic studies were performed on the same device, using the same protocol by one experienced pediatric cardiologist (R.P.). The obvious limitations of this research are the lack of a control group and the cross-sectional character of the study. All inflammatory markers, including MNR, were evaluated on a single measurement and obviously could have also been influenced by conditions other than PH. This last limitation precludes concluding the causal relationship between inflammatory markers (e.g., MNR) and the formation of left ventricular hypertrophy. Although we performed a thorough interview and physical examination, we cannot rule out other hidden sources of inflammation, e.g., periodontal disease and parasitosis—the patients were not examined for these entities.

## 5. Conclusions

Primary hypertension is currently considered a multisystemic disease, and activation of the immune system is involved in its pathogenesis. In up to half of children at the time of diagnosis of primary hypertension, hypertension-mediated organ damage is already found, most often left ventricular hypertrophy. Our cross-sectional study revealed left ventricular hypertrophy in about one-third of patients. In our group, we analyzed many markers of subclinical inflammation, including hs-CRP, interleukin 18, and markers derived from complete blood count. We showed that the monocyte-to-neutrophil ratio was the most important immunological biomarker of left ventricular hypertrophy, and various statistical methods demonstrated its significance. We see the need for further research on this new, simple, and promising marker in children with primary hypertension.

## Figures and Tables

**Table 1:** Clinical and biochemical data of the study group.

Parameter	Value ± SD [Quartiles]
Number of patients (*n*)	34
Boys/girls (*n*, %)	26/8 (76%/24%)
Age (years)	15.1 ± 2.1 (from 10.9 to 17.9) [13.8–16.8]
Duration of gestation (weeks)	38.9 ± 2.5 [38–40]
Birth weight (g)	3079 ± 696 [2620–3685]
Duration of hypertension (months)	13.2 ± 13.0 [3–24]
BMI Z-score	1.49 ± 0.82 [0.99–2.01]
Overweight patients (BMI 85–95 c) (*n*, %)	7 (21%)
Obese patients (BMI > 95 c) (*n*, %)	17 (50%)
GFR (mL/min/1.73 m^2^)	98.2 ±19.8 [85.4–110.8]
Total cholesterol (mg/dL)	166.7 ± 25.3 [150–181]
LDL-cholesterol (mg/dL)	93.2 ± 24.0 [72–109]
HDL-cholesterol (mg/dL)	50.5 ± 14.6 [40–60]
Triglycerides (mg/dL)	114.0 ± 58.9 [65–145]
Uric acid (mg/dL)	6.3 ± 1.8 [5.2–7.2]
25OHD (ng/mL)	18.4 ± 7.9 [12.2–21.5]
ACR (mg/g)	18.9 ± 58.0 [5.3–12.8]

SD—standard deviation, BMI—body mass index, GFR—glomerular filtration rate, LDL—low-density lipoprotein, HDL—high-density lipoprotein, 25OHD—25-hydroxyvitamin D, ACR—urinary albumin-to-creatinine ratio.

**Table 2:** Inflammatory markers in the study group.

Parameter	Value ± SD [Quartiles]
High-sensitivity C-reactive protein (hs-CRP) (mg/L)	4.9 ± 5.4 [1.5–6.9]
Interleukin-18 (IL-18) (pg/mL)	81.5 ± 80.8 [34.5–110.1]
Neutrophil count (1000/µL)	4.0 ± 1.3 [3.1–4.8]
Monocyte count (1000/µL)	0.6 ± 0.2 [0.5–0.7]
Lymphocyte count (1000/µL)	2.5 ± 0.7 [2.0–2.8]
Platelet count (1000/µL)	270.2 ± 56.7 [233–300]
Mean platelet volume (fL)	10.4 ± 1.4 [9.8–11.4]
Neutrophil-to-lymphocyte ratio (NLR)	1.7 ± 0.5 [1.4–1.9]
Platelet-to-lymphocyte ratio (PLR)	115.6 ± 31.5 [88.9–133.6]
Monocyte-to-lymphocyte ratio (MLR)	0.3 ± 0.1 [0.2–0.3]
Monocyte-to-neutrophil ratio (MNR)	0.2 ± 0.1 [0.1–0.2]
Platelet-to-mean platelet volume ratio (PMPVR) (10^12^/fL)	26.5 ± 7.1 [21.0–31.0]

**Table 3:** Blood pressure in the study group.

Parameter	Value ± SD [Quartiles]
Office systolic blood pressure (mm Hg)	141.7 ± 9.8 [133–148]
Office systolic blood pressure Z-score	2.3 ± 0.8 [1.9–3.0]
Office diastolic blood pressure (mm Hg)	84.2 ± 10.7 [78–94]
Office diastolic blood pressure Z-score	2.6 ± 1.4 [1.6–3.8]
Office pulse pressure (mm Hg)	57.4 ± 8.8 [51–63]
24 h ABPM systolic blood pressure (mm Hg)	134.5 ± 5.1 [132–139]
24 h ABPM systolic blood pressure Z-score	2.3 ± 0.8 [1.8–2.6]
24 h ABPM diastolic blood pressure (mm Hg)	73.1 ± 6.8 [70–76]
24 h ABPM diastolic blood pressure Z-score	0.9 ±1.2 [0.4–1.4]
24 h ABPM mean blood pressure (mm Hg)	92.7 ± 5.9 [89–96]
24 h ABPM mean blood pressure Z-score	1.6 ± 1.2 [0.8–1.9]
24 h ABPM systolic blood pressure load (%)	56.9 ± 17.9 [44–69]
24 h ABPM diastolic blood pressure load (%)	26.2 ± 20.8 [14–34]
ABPM systolic blood pressure dipping (%)	11.3 ± 5.6 [7.2–14.9]
ABPM diastolic blood pressure dipping (%)	15.9 ± 7.9 [10.5–20.0]

ABPM—ambulatory blood pressure monitoring.

**Table 4:** Echocardiographic parameters in the study group.

Parameter	Value ± SD [Quartiles]
IVSd (mm)	0.82 ± 0.16 [0.70–0.93]
IVSd Z-score	1.1 ± 0.9 [0.5–1.9]
LVEDd (mm)	5.02 ± 0.57 [4.70–5.40]
LVEDd Z-score	−0.9 ± 1.0 [−1.6–−0.1]
LVPWd (mm)	0.83 ± 0.17 [0.70–0.96]
LVPWd Z-score	0.5 ± 0.9 [0.0–1.1]
LVM (g)	146.3 ± 46.6 [116.6–188.1]
LVM for lean body mass Z-score	0.17 ± 1.35 [−0.93–1.32]
LVM for height Z-score	−0.40 ± 1.52 [−1.43–0.99]
LVMI (g/m^2^)	75.7 ± 21.2 [59.0–90.0]
LVMI (g/m^2.7^)	34.1 ± 9.0 [27.0–42.0]
LVMI (g/m^2.16^)	44.2 ± 11.9 [34.0–54.0]
RWT (2 x LVPWd/LVEDd)	0.34 ± 0.07 [0.30–0.39]
RWT (2 x IVSd/LVEDd)	0.33 ± 0.06 [0.27–0.37]
RWT (IVSd + LVPWd)/LVEDd)	0.33 ± 31.6 [64–103]

IVSd—the interventricular septum thickness at end diastole, LVEDd—left ventricular inner dimension at end diastole, LVPWd—left ventricular posterior wall at end diastole, LVM—left ventricular mass, LVMI—left ventricular mass index, RWT—relative wall thickness.

**Table 5:** Clinical and biochemical parameters, blood pressure, and inflammatory markers in patients without and with left ventricular hypertrophy.

Analyzed Parameter	Normal Left Ventricular Mass Value ± SD [Quartiles]	Left Ventricular Hypertrophy Value ± SD [Quartiles]	*p*
Number of patients	22	12	-
Boys/girls (*n*, %)	16/6	10/2	0.681
Age (years)	15.1 ± 2.0 [14.1–16.8]	15.0 ± 2.2 [13.7–16.7]	0.524
Gestation (weeks)	38.5 ± 2.9 [37–40]	39.9 ± 0.8 [40–40]	0.268
Birth weight (g)	3058 ± 760 [2540–3770]	3173 ± 389 [2840–3600]	0.905
Duration of PH (months)	13.5 ± 14.0 [2–24]	12.8 ± 11.7 [3–21]	0.943
BMI Z-score	1.46 ± 0.82 [0.93–1.84]	1.54 ± 0.87 [1.00–2.36]	0.430
Total cholesterol (mg/dL)	166.4 ± 22.0 [151–177]	167.1 ± 31.6 [144–198]	0.418
LDL-cholesterol (mg/dL)	93.9 ± 20.8 [75–104]	92.0 ± 30.0 [68–127]	0.462
HDL-cholesterol (mg/dL)	52.1 ± 16.3 [38–61]	47.7 ± 10.7 [43–54]	0.204
Triglycerides (mg/dL)	102.2 ± 53.3 [63–119]	135.6 ± 64.7 [91–160]	0.126
Uric acid (mg/dL)	6.4 ± 1.8 [5.2–7.8]	6.2 ± 1.8 [5.1–6.6]	0.751
25OHD (ng/mL)	18.3 ± 7.2 [13.7–21.3]	18.5 ± 9.3 [11.1–23.3]	0.182
ACR (mg/g)	24.5 ± 72.0 [5.3–14.2]	8.7 ± 4.7 [5.3–12.1]	0.928
Office SBP (mm Hg)	143.1 ± 10.7 [138–152]	138.9 ± 7.6 [133–143]	0.238
Office SBP Z-score	2.4 ± 0.9 [1.6–3.2]	2.1 ± 0.6 [1.9–2.3]	0.255
Office DBP (mm Hg)	86.5 ± 11.6 [80–97]	80.1 ± 7.8 [73–84]	0.096
Office DBP Z-score	2.9 ± 1.6 [2.0–4.3]	2.0 ± 1.0 [1.1–2.6]	0.091
Office PP (mm Hg)	56.6 ± 9.3 [50–60]	58.8 ± 7.9 [55–66]	0.495
24 h ABPM SBP (mm Hg)	134.2 ± 5.2 [132–139]	135.0 ± 5.2 [132–138]	0.732
24 h ABPM SBP Z-score	2.3 ± 0.8 [1.8–2.6]	2.3 ± 0.8 [1.8–2.6]	0.349
24 h ABPM DBP (mm Hg)	73.7 ± 7.4 [71–77]	72.0 ± 5.9 [69–75]	0.489
24 h ABPM DBP Z-score	0.9 ±1.2 [0.4–1.4]	0.9 ±1.2 [0.4–1.4]	0.495
24 h ABPM MAP (mm Hg)	93.3 ± 5.7 [90–96]	91.7 ± 6.3 [87–96]	0.457
24 h ABPM MAP Z-score	1.6 ± 1.2 [0.8–1.9]	1.6 ± 1.2 [0.8–1.9]	0.601
24 h ABPM SBP load (%)	58.8 ± 19.5 [44–73]	53.3 ± 14.8 [47–61]	0.377
24 h ABPM DBP load (%)	29.2 ± 20.8 [14–34]	20.5 ± 16.3 [11–22]	0.248
ABPM SBP dipping (%)	11.9 ± 6.1 [7.6–15.1]	10.4 ± 4.8 [4.7–14.8]	0.705
ABPM DBP dipping (%)	17.0 ± 8.4 [11.0–20.0]	13.8 ± 6.9 [8.6–20.0]	0.449
hs-CRP (mg/L)	4.4 ± 4.6 [1.2–6.8]	5.8 ± 6.8 [1.6–7.2]	0.449
IL-18 (pg/mL)	91.2 ± 93.6 [34.5–136.3]	63.5 ± 47.9 [19.2–94.2]	0.552
Neutrophil count (1000/µL)	4.1 ± 1.2 [3.4–4.8]	3.7 ± 1.6 [2.8–4.4]	0.360
Monocyte count (1000/µL)	0.6 ± 0.2 [0.5–0.7]	0.6 ± 0.3 [0.5–0.7]	0.759
Lymphocyte count (1000/µL)	2.3 ± 0.4 [2.0–2.7]	2.7 ± 1.0 [1.9–3.6]	0.843
Platelet count (1000/µL)	276.0 ± 65.9 [233–318]	259.5 ± 34.1 [230–284]	0.427
MPV (fL)	10.1 ± 1.4 [9.2–11.1]	11.0 ± 1.3 [10.7–11.9]	0.065
NLR	1.8 ± 0.5 [1.5–2.1]	1.4 ± 0.4 [1.1–1.8]	0.043
PLR	119.8 ± 30.1 [92.0–140.2]	108.0 ± 34.1 [70.2–131.9]	0.302
MLR	0.3 ± 0.1 [0.2–0.4]	0.2 ± 0.1 [0.2–0.3]	0.608
MNR	0.1 ± 0.1 [0.1–0.2]	0.2 ± 0.0 [0.2–0.2]	0.042
PMPVR (10^12^/fL)	27.9 ± 8.0 [22.5–33.0]	23.9 ± 4.4 [20.8–26.1]	0.112

SD—standard deviation, BMI—body mass index, LDL—low-density lipoprotein, HDL—high-density lipoprotein, 25OHD—25-hydroxyvitamin D, ACR—urinary albumin-to-creatinine ratio, SBP—systolic blood pressure, DBP—diastolic blood pressure, MAP—mean arterial pressure, SBPL—systolic blood pressure load, DBPL—diastolic blood pressure load, hs-CRP—high-sensitivity C-reactive protein, IL-18—interleukin 18, MPV—mean platelet volume, NLR—neutrophil-to-lymphocyte ratio, PLR—platelet-to-lymphocyte ratio, MLR—monocyte-to-lymphocyte ratio, MNR—monocyte-to-neutrophil ratio, PMPVR—platelet-to-mean platelet volume ratio.

**Table 6:** Diagnostic accuracy of mean platelet volume, neutrophil-to-lymphocyte ratio, and monocyte-to-neutrophil ratio in predicting left ventricular hypertrophy.

Parameter	Area Under the Curve (95% CI)	*p*	Inflammatory Marker Cut-Off Value	Sensitivity	Specificity	ACC
MPV	0.729 (0.546–0.912)	0.014	10.6	0.833	0.682	0.735
NLR	0.697 (0.509–0.885)	0.040	1.321	0.955	0.417	0.765
MNR	0.701 (0.525–0.877)	0.025	0.163	0.750	0.682	0.706

CI—confidence interval, ACC—accuracy, MPV—mean platelet volume, NLR—neutrophil-to-lymphocyte ratio, MNR—monocyte-to-neutrophil ratio.

## Data Availability

Data used to support the findings of this study are included within the Supplementary Materials files, Table S2 (Table S2 patient_data.xlsx).

## Supplementary Materials

The following supporting information can be downloaded at: https://www.mdpi.com/article/10.3390/jcm14113896/s1, Table S1: Correlations between body mass index, high-sensitivity C-reactive protein and echocardiographic parameters in the studied children. Table S2: Patient data (Table S2 patient_data.xlsx).
