# Supplementary material for: Monocyte-to-Neutrophil Ratio as an Immunological Marker of Left Ventricular Hypertrophy in Children with Primary Hypertension
Source: J Clin Med. 2025 Jun 1;14(11):3896. doi: 10.3390/jcm14113896 (PMC12156006; doi:10.3390/jcm14113896)
Supplement: Supplementary file 1 [file jcm-14-03896-s001.zip › Table S1.pdf]

Supplementary Table 1

Correlations between body mass index, high-sensitivity C-reactive protein and echocardiographic parameters in the studied children.

	BMI [kg/m^2]	BMI Z-score
hsCRP [mg/L]	R=0.005, P=0.979	R=0.035, P=0.846
IVSd [mm]	R=0.231, P=0.189	R=0.213, P=0.226
IVSd Z-score	R=-0.006, P=0.975	R=0.021, P=0.917
LVEDd [mm]	R=0.135, P=0.447	R=-0.001, P=0.994
LVEDd Z-score	**R=-0.463, P=0.015**	**R=-0.540, P=0.004**
LVPWd [mm]	**R=0.362, P=0.035**	R=0.289, P=0.098
LVPWd Z-score	R=-0.092, P=0.650	R=-0.097, P=0.630
LVM [g]	R=0.291, P=0.095	R=0.180, P=0.309
LVM for lean body mass Z-score	R=-0.128, P=0.492	R=-0.138, P=0.459
LVM for height Z-score	R=0.209, P=0.236	R=0.154, P=0.386
LVMI [g/m^2]	R=-0.107, P=0.547	R=-0.173, P=0.327
LVMI [g/m$^{2.7}$]	R=0.181, P=0.305	R=0.135, P=0.448
LVMI [g/m$^{2.16}$]	R=0.221, P=0.209	R=0.152, P=0.390
RWT [2 x LVPWd / LVEDd]	R=0.235, P=0.180	R=0.248, P=0.158
RWT [2 x IVSd / LVEDd]	R=0.142, P=0.422	R=0.207, P=0.240
RWT [(IVSd + LVPWd) / LVEDd]	R=0.209, P=0.236	R=0.249, P=0.156

hs-CRP – high-sensitivity C-reactive protein, IVSd - the interventricular septum thickness at end diastole, LVEDd - left ventricular inner dimension at end diastole, LVPWd - left ventricular posterior wall at end diastole, LVM – left ventricular mass, LVMI – left ventricular mass index, RWT – relative wall thickness
